# First Finding of a Methicillin-Resistant *Staphylococcus aureus* (MRSA) t304/ST6 from Bovine Clinical Mastitis

**DOI:** 10.3390/antibiotics11101393

**Published:** 2022-10-12

**Authors:** Desiree Corvera Kløve, Vibeke Frøkjær Jensen, Lærke Boye Astrup

**Affiliations:** Center for Diagnostics, Technical University of Denmark, Kemitorvet, 2800 Kgs. Lyngby, Denmark

**Keywords:** *Staphylococcus aureus*, bovine mastitis, antimicrobial resistance (AMR), methicillin-resistant *Staphylococcus aureus* (MRSA), antimicrobial susceptibility testing (AST), minimum inhibitory concentration (MIC), whole-genome sequencing (WGS)

## Abstract

The emergence of methicillin-resistant *Staphylococcus aureus* (MRSA) comprises a global threat to humans and animals. Here, we report and characterize the MRSA t304/ST6 variant which, to our knowledge, represents the first case found in bovine clinical mastitis. In general, the MRSA t304/ST6 variant is rarely described in livestock, contrary to humans where it is widely recognized. Phenotypic and genotypic resistance profiling showed that the bovine-MRSA t304/ST6 isolate expressed low susceptibility toward cefoxitin (MIC_cefoxitin_ = 16 µg/mL) and carried the *mecA* resistance gene in the SCC*mec* IVa. The bovine-MRSA t304/ST6 isolate carried a plasmid similar to that which has been frequently observed among human-MRSA t304/ST6 isolates in Denmark (GenBank accession no. NZ_CP047022). In addition, a Staphylococcus prophage 3 (ϕSA3) was detected, encoding an immune evasion cluster (IEC) of putative virulence genes associated with human host-specificity (*sea*, *sak*, and *scn*). Taken together, these findings suggest that the MRSA t304/ST6 found in this study represents a recent host-jump event, with human to cow transmission. This study emphasizes the importance of and the need for performance of antimicrobial resistance surveillance among bovine mastitis pathogens, including *S. aureus* and MRSA.

## 1. Introduction

*Staphylococcus aureus* causes severe infections in both humans and animals and is a major cause of bovine mastitis [1]. Internationally, treatment of bovine mastitis caused by *S. aureus* relies on β-lactams (e.g., penicillins and cephalosporins) [2,3]. Nevertheless, some countries, such as Denmark, recommend only narrow-spectrum penicillin as the drug of first choice [4,5]. Accordingly, the increasing occurrence of methicillin-resistant *S. aureus* (MRSA), that is resistant to all penicillins and cephalosporins, is of concern. To enable strategies to combat antimicrobial resistance (AMR), it is crucial to surveil the development of resistant bacteria, including bovine mastitis pathogens, such as *S. aureus*. Surveillance creates data on the prevalence, distribution, and co-factors of AMR, thus enabling analysis on the correlations between AMR and, e.g., antibiotic consumption, inter-host transmissions, and so on. Therefore, many countries have established AMR surveillance programs. In Denmark, however, the national AMR surveillance program does not include mastitis pathogens. Systematic screening for MRSA among dairy cows is carried out, but the procedure is based on nasal swab samples. Hence, even though mastitis is one of the diseases causing the most antibiotic consumption in adult cattle we know very little on the occurrence of AMR in bovine mastitis [6,7]. As such, knowledge on AMR among Danish mastitis pathogens relies mainly on research performance. One such research study on 24 *S. aureus* isolates from bovine clinical mastitis collected in 2018–2019, found a single isolate resistant towards cefoxitin (2nd-generation cephalosporin), suggesting it was an MRSA [8]. The current paper presents a molecular follow-up study on that cefoxitin-resistant *S. aureus* isolate, based on whole-genome sequencing (WGS) analysis. Hence, the first objective of the present study was to clarify if the isolate was truly an MRSA through genotypic resistance profiling. The second objective was to characterize the genotype by *Staphylococcus aureus* Protein A (*spa*) typing and multi-locus sequence typing (MLST). The genotype was further characterized with a special focus on mobile genetic elements (MGEs) and virulence genes.

## 2. Results

### 2.1. Resistance Profiling

The cefoxitin-resistant *S. aureus* isolate was discovered and tested for antimicrobial susceptibility toward 14 agents as part of a previous study [8]. The isolate was originally found in mixed culture with *E. faecalis*.

The minimum inhibitory concentration (MIC) values for each agent are presented in Table 1, as they were not previously provided. The cefoxitin-resistant *S. aureus* isolate was found resistant towards cefoxitin (MIC = 16 µg/mL) and penicillin (MIC > 16 µg/mL). The MIC values for the isolate were low for the remaining 12 agents tested.

The cefoxitin-resistant *S. aureus* isolate was whole-genome sequenced to confirm if it was an MRSA (assembly metrics are given in Table S1). For this analysis, the assembled genome was screened for the presence of resistance genes.

Two resistance genes were found, *blaZ* and *mecA*, both conferring β-lactam resistance (Table S2). The presence of the *mecA* gene confirmed that the cefoxitin-resistant *S. aureus* isolate was an MRSA. Both resistance genes were found with > 99% identity and 100% coverage in the blast toward the corresponding reference genes in the ResFinder database. Additionally, Staphylococcal cassette chromosome *mec* (SCC*mec*) typing revealed that the *mecA* gene was an element of the SCC*mec* type Iva.

### 2.2. Genotypic Characterization of Cefoxitin-Resistant S. aureus Isolate

Upon MRSA confirmation based on resistance profiling, additional characteristics of the isolate were explored. Performance of *spa* typing showed that the isolate belonged to t304, and MLST revealed that it was a ST6. Accordingly, in the following sections, the cefoxitin-resistant *S. aureus* isolate will be referred to as the bovine-MRSA t304/ST6 isolate.

Investigation of present virulence genes in the bovine-MRSA t304/ST6 isolate was based on the VirulenceFinder and VFDB databases. The analysis revealed a total number of 73 putative virulence genes, which are summarized in Table S3. These putative virulence genes indicated different virulence factors of the bovine-MRSA t304/ST6 isolate; for instance, host immune evasion is shown by the presence of several genes encoding for leukotoxins. Examples of such found putative genes were *lukE* and *lukD* (encoding LukED), *hlgA*, *hlgB*, and *hlgC* (encoding γ-Hemoylsin (Hlg)), and the *lukF-PV* subunit F of the Panton-Valentine Leukocidin (PVL). Additional putative virulence genes involved in immune evasion found were *sea*, *sak*, and *scn* forming an immune evasion cluster (IEC) (Table S3). Using PHASTER, the identified IEC was located at a Staphylococcus prophage 3 (ϕSA3) (GenBank accession no. NC_048644.1) along with the β-hemolysin gene (*hlb*). Furthermore, a putative virulence gene of the superantigen von Willebrand binding protein (vWbp) was found.

Finally, application of MobileElementFinder together with PlasmidFinder revealed that the bovine-MRSA t304/ST6 isolate carried a plasmid containing the rep16 and rep5 plasmid replicon genes and the *blaZ* resistance gene.

## 3. Discussion

The present study comprises a molecular follow-up study on a cefoxitin-resistant *S. aureus* isolate from bovine clinical mastitis discovered in a previous study [8]. The results from molecular characterization based on WGS analysis showed that the cefoxitin-resistant isolate was indeed an MRSA, that it belonged to the variant t304/ST6, and that it carried several MGEs and putative virulence genes.

The MRSA verification was based on the detection of the *mecA* gene in the SCC*mec* type IVa. From *spa* typing and MLST analysis, it was found that the MRSA isolate was a t304/ST6, which is an MRSA variant rarely described in animals. To our knowledge, the present study represents the first description of an MRSA t304/ST6 isolated from bovine clinical mastitis. However, the t304/ST6 variant has been previously described once in bovine mastitis but in a case of methicillin-susceptible *Staphylococcus aureus* (MSSA) [10,11]. Contrary, the t304/ST6 variant is generally considered a community-associated (CA) MRSA, which seems to be more described- and disseminated among humans [11,12]. In Denmark, the MRSA t304/ST6 variant was first detected in 2011 where it caused a long-term outbreak in a neonatal intensive care unit in Copenhagen [13]. A recent study presumes that this variant originates from the Middle East and has been introduced to the Danish community by human refugees [11]. Indeed, the MRSA t304/ST6 variant has shown success at spreading in human populations, as the occurrence of this variant has increased during the last decade in several northern European countries, including Denmark [11,14]. Overall, in Denmark, the number of new human MRSA cases has been increasing up to 2017. In 2017, only six years after its introduction, the t304/ST6 variant was the second most common MRSA variant, while the livestock associated (LA)-MRSA ST398 remained the most widespread [14]. However, despite the rise in new human MRSA cases, Denmark is generally considered a low prevalence country regarding MRSA. This is also in accordance with the findings from the national systematic screening of MRSA among dairy cattle in Denmark [15]. However, as the current MRSA screening relies on nasal swabs, data on MRSA occurrence from other sources of the cows (e.g., the udder) is currently missing or scarce.

The dominant LA-MRSA ST398 variant is primarily associated with pig farming and has gone through species host-jump events (i.e., bacterial adaption to different host species) [16,17]. Mobile genetic elements (e.g., plasmids and phages) are demonstrated to play a crucial role in regard to host-jump activities. This is because MGEs are often reservoirs of virulence and resistance genes, influencing the pathogenic features of how the bacteria causes host infection. In addition, MGEs are capable of easy movement within- and across bacterial genomes. Thereby, MGEs cause gene gain and loss, and they can subsequently lead to genetic adaption to new niches [18]. Evolutionary studies of *S. aureus* strains have revealed that some MGEs and virulence genes are host-specific [19,20]. To date, several of such bovine-specific virulence factors are described, for instance some leukotoxins, which are associated with lytic activity against white blood cells. The vWbp and the LukMF leukotoxin are examples, which are both involved in immune evasion, and comprise some of the best characterized virulence genes with bovine host-specificity [19,20]. In the present study, we found a putative virulence gene corresponding to vWbp (Table S3) but no genes encoding the LukMF leukotoxin (*lukM* or *lukF*). Putative virulence genes of LukED (encoded by *lukE* and *lukD*) and Hlg (encoded by *hlgA*, *hlgB*, and *hlgC*) was however found (Table S3). These leukotoxins have been previously observed among bovine *S. aureus* strains [20,21] but are presumably associated with human infection [22]. The PVL leukotoxin (encoded by *lukF-PV* and *lukS-PV*) is one of the best studied *S. aureus* virulence factors, which is also commonly associated with CA-MRSA [23,24]. In the present study, a putative virulence gene of the subunit F of PVL (*lukF-PV*) was found, which could indicate a recent gene loss of the subunit S (*lukS-PV*). Nonetheless, due to the missing *lukS-PV* gene, the bovine-MRSA t304/ST6 in this study would be classified as PVL-negative. This finding correlates with results from a recent study by Bartels and associates covering human-MRSA t304/ST6 isolates from northern European countries, mainly Denmark, which found that 95% of all human-MRSA t304/ST6 were PVL-negative [11]. In the same study, 94% of the human-MRSA t304/ST6 carried the *mecA* resistance gene in the SCC*mec* IVa. The same SCC*mec* IVa was also demonstrated for the present bovine-MRSA t304/ST6 isolate. Furthermore, Bartels and associates identified a novel plasmid (NCBI Accession number NZ_CP047022) carrying two plasmid replication genes (rep16 and rep5), and the *blaZ* resistance gene in 79% of the human-MRSA t304/ST6 isolates. This plasmid was also detected in the present bovine-MRSA t304/ST6 isolate.

Altogether, these findings imply a common origin of the MRSA t304/ST6 variant. In addition, they suggest that the bovine-MRSA t304/ST6 isolate had human origin and was previously transmitted to the cow through a recent host-jump event. This hypothesis is supported further by the discovery of a ΦSa3, which was carrying an IEC of putative virulence genes (*sea*, *sak*, and *scn*) associated with human host-specificity [19,24]. Moreover, the *scn* gene is generally included as a marker for human adaption in the current test-setup for detection of MRSA among humans in Denmark [14]. Transmission of MRSA between humans and cows was previously suspected. For instance, in Sweden, an MRSA outbreak at a dairy herd persisted for at least two years and was assumingly due to initial introduction by the farmer [25].

The present study covers a single MRSA t304/ST6 isolate from a cow with clinical mastitis, having visual clinical symptoms on two glands of the udder. The milk samples from these glands both contained *E. faecalis*, one in pure culture and one in mixed culture together with the described MRSA t304/ST6 isolate. One can never fully eliminate the possibility of the current finding being due to a human contamination of the milk or udder. Such contamination could occur during milking, milk sampling, or laboratory handling. However, the milk was sampled aseptically and processed in a veterinary clinic and at a professional quality-assured lab (Center for Diagnostics, Technical University of Denmark (CfD, DTU)) only. Furthermore, the finding of two milk samples without contamination and from a cow with visual clinical signs in both glands suggests that the bacteria arise from the cow and not from human contamination. Moreover, the presence of the bovine-specific vWbp further contributes to opposing the possibility of human contamination of the milk sample [20].

The WHO One Health approach highlights the importance of considering health of humans and animals together and as interlinked [26]. This also applies when studying the occurrence and development of AMR, as AMR traits may easily transfer between host species through MGEs and/or host-jump events. Therefore, it is crucial not only to monitor AMR among pathogens in humans but in animals as well. The bovine-MRSA t304/ST6 from the present study was originally identified as part of a previous study covering AST and AMR occurrence among 24 *S. aureus* isolates from bovine clinical mastitis in Denmark [8]. The isolates in that study represented a randomized population, as they originated from milk samples collected from different dairy herds by different veterinarians and were submitted for diagnostic analysis at CfD, DTU during 2018–2019 as part of a larger screening project and without any selection criteria. The present study confirms the single found cefoxitin-resistant isolate to be an MRSA, which means that 1/24 *S. aureus* isolates were MRSA positive in the previous study [8]. Consequently, one may speculate if the occurrence of MRSA from bovine clinical mastitis in Denmark is currently underreported.

In conclusion, we here report the first finding and description of the CA-MRSA t304/ST6 variant in bovine clinical mastitis. Overall, the genotypic characterization revealed the presence of MGEs and virulence genes that have been previously associated with *S. aureus* and MRSA human host-specificity. However, a putative virulence gene of vWbp, which is generally linked to bovine host-specificity [20], was detected as well. Altogether, findings from this study may demonstrate a previous host-jump case, with human to cow transmission, and successful niche adaption to bovine hosts. Based on the current results, we emphasize the importance of performing AMR surveillance on mastitis pathogens, including *S. aureus* and MRSA.

## 4. Materials and Methods

### 4.1. Isolate Identification and Antimicrobial Susceptibility Testing

The cefoxitin-resistant *S. aureus* isolate was identified as part of a large national research study on antimicrobial resistance that was conducted in 2018–2019 in collaboration with 11 veterinary clinics [8]. In brief, veterinarians collected milk samples from cows with clinical mastitis. The number of milk samples collected from each cow differed according to how many glands on which clinical symptoms appeared. After conducting their own in-house diagnostics, the veterinary clinics provided milk samples to CfD, DTU for laboratory examination and performance of AST. When obtained at CfD, DTU the milk samples were cultured on blood agar (5% calf blood, SSI Diagnostica A/S, Hillerød, Denmark) and incubated overnight at 37 °C, then subcultured on blood agar and incubated overnight again. Pure subcultures were identified by matrix-assisted laser desorption/ionization time of flight mass spectroscopy (MALDI-TOF MS) [27]. Isolates from all samples with one or two pathogen species were stored at −80 °C in LB bouillon with 15% glycerol for further analysis. All samples containing more than two pathogen species were discharged as contaminated [28].

A subset of the identified pathogens, including *S. aureus*, were selected for AST. The MICs were determined by broth dilution method using SensiTitre (SensiTitre; TREK Diagnostic Systems). The MIC panel used, DKVP, comprised the panel routinely applied at CfD, DTU for AST performance of veterinary Gram-positive bacteria [29]. The panel contained 14 agents which were either relevant for veterinary usage or for one-health surveillance purposes, respectively. The test agents and corresponding test ranges are provided in Table 1. The obtained MIC values were interpreted by usage of epidemiological cutoff values (ECOFFs) set by EUCAST [9] given in Table 1. In total, 24 *S. aureus* isolates were MIC-tested.

### 4.2. Whole-Genome Sequencing and Bioinformatic Analyses

The cefoxitin resistant *S. aureus* isolate was collected in 2019 and was investigated for being an MRSA as follows. DNA extraction, purification, and whole-genome sequencing was outsourced to Novogene (Novogene (Cambridge, UK) Co., Ltd.). Briefly, DNA was extracted by usage of an AllPrep DNA/RNA kit (Qiagen, Hilden, Germany) and subsequently paired-end sequenced applying Illumina’s NovaSeq 6000 platform with a read length of 2 × 150 bp.

Quality assessment of the raw reads was analyzed in FastQC (v.0.11.9). Assembly was carried out using the SPAdes pipeline (v.3.13.1) with the command line “-k 21,33,55,77--careful” [30]. The quality of the assembled genome was examined by QUAST (v.5.0.2) [31]. Prokka (v. 1.14.6) was used for gene annotation [32]. For subtyping and identification of genomic content mainly services of the Center for Genomic Epidemiology (cge), DTU was applied, in every case, with default thresholds. Subtyping involved *spa* typing by SPAtyper (v1.0) [33], MLST classification by MLST (v. 2.0) [34], and SCCmec identification by SCCmecFinder (v.1.2) [35]. Detection of MGEs was based on MobileElementFinder (v. 1.0.3) [36], PlasmidFinder (v. 2.0) [37], and PHASTER [38]. Finally, the prevalence of putative resistance and virulence genes was explored using the Abricate package [39] together with the Resfinder [40] and VFDB [41] databases, as well as VirulenceFinder (v. 2.0) [42,43]. Thresholds were set to 80% for identify and coverage, respectively. Furthermore, the genome was blasted against the plasmid sequence (NCBI Accession number NZ_CP047022) using blastn.

## Figures and Tables

**Table 1:** MIC values of cefoxitin-resistant *S. aureus* isolate from a case of bovine clinical mastitis.

Agent	Test Range (µg/mL)	MIC Value (µg/mL)	(t)ECOFF (µg/mL)
Cefoxitin (FOX)	0.5–32	16	4
Chloramphenicol (CHL)	2–64	8	16
Ciprofloxacin (CIP)	0.125–8	0.25	1
Erythromycin (ERY)	0.25–16	0.5	1
Florfenicol (FFN)	1–64	8	(8)
Gentamicin (GEN)	0.25–16	≤0.25	2
Penicillin (PEN)	0.06–16	>16	0.125 *
Spectinomycin (SPE)	16–256	64	(128)
Streptomycin (STR)	4–64	8	16
Sulphamethoxazole (SMX)	32–512	64	(128)
Tetracycline (TET)	0.5–32	1	1
Tiamulin (TIA)	0.25–32	1	(2)
Trimethoprim (TMP)	0.5–32	2	2
TMP + SMX (STX)	0.25–16	≤0.25	(0.25)

Epidemiological Cut-Off (ECOFF) values provided in brackets are tentative (t)ECOFFs [9]. * ECOFF value adapted from Benzylpenicillin.

## Data Availability

Not applicable.

## Supplementary Materials

The following supporting information can be downloaded at: https://www.mdpi.com/article/10.3390/antibiotics11101393/s1, Table S1: Assembly metrics of MRSA t304/ST6 genome, Table S2: Presence of resistance genes in a MRSA t304/ST6 isolate from a case of bovine clinical mastitis, Table S3: Presence of putative virulence genes in a MRSA t304/ST6 isolate from a case of bovine clinical mastitis.
